# Ligamentotaxis-Based Dynamic External Fixation for Comminuted Intra-articular Fractures of the Proximal Phalanx Base of the Thumb: A Report of Two Cases

**DOI:** 10.7759/cureus.116681

**Published:** 2026-09-21

**Authors:** Mohammad A Ali

**Affiliations:** 1 Department of Trauma and Orthopedic, Arm and Hand Microsurgery Unit, Medcare Orthopaedic and Spine Hospital, Dubai, ARE

**Keywords:** dynamic external fixators, finger phalanges, ligamentotaxis, phalangeal fractures, thumb injuries

## Abstract

Complex intra-articular fractures of the proximal phalanx of the thumb present significant management challenges due to comminution, displacement, and the thumb’s essential functional role. Traditional fixation techniques may risk soft-tissue injury or fail to provide stable conditions for early mobilization. Dynamic external fixation (DEF) using ligamentotaxis offers a minimally invasive alternative, though its use in comminuted proximal phalanx fractures of the thumb remains sparsely reported. This report describes two male patients with displaced, comminuted intra-articular fractures of the base of the proximal phalanx, resulting from a crush injury and a fall from height. Computed tomography imaging confirmed substantial articular displacement in both cases. Both patients underwent mini-open reduction under fluoroscopic guidance, followed by placement of a ligamentotaxis-based DEF. This technique restored articular congruity while minimizing soft-tissue disruption. Both patients recovered uneventfully with preserved neurovascular function, maintained fracture reduction, near-normal range of motion, grip strength recovery of 90-95%, full thumb opposition, and return to work within four to five weeks. These cases support ligamentotaxis-based DEF as a feasible option for comminuted intra-articular fractures of the proximal phalanx base of the thumb.

## Introduction

The thumb, with its unique ability to contact the other fingers, is fundamental to effective hand function. Without this capability, essential tasks such as writing, manipulating tools, or even maintaining a secure grip would be markedly impaired [1]. Its flexion range is relatively limited, but joint stability in both flexion and extension is essential to resist radial and ulnar deviation during pinch and grasp. Functionally, the thumb metacarpophalangeal (MCP) joint behaves like a hinge, emphasizing stability rather than mobility [2]. Consequently, fractures involving the base of the proximal phalanx can substantially compromise thumb mechanics and hand function, particularly when the articular surface is involved.

Fractures involving the base of the proximal phalanx may present as transverse extra-articular fractures; however, the fracture line may extend into the articular surface or propagate proximally from the shaft toward the base. In intra-articular fractures, the degree of joint incongruity is a key determinant in deciding the need for surgical intervention [3]. Restoration of articular alignment while preserving joint mobility is therefore essential for optimal functional recovery.

Management of these fractures remains challenging because of the complex anatomy and biomechanics of the thumb [4]. Treatment decisions depend on several factors, including fracture location, degree of displacement or angulation, presence of rotational deformity, and associated soft-tissue injury. Inadequate or inappropriate management can result in complications such as malunion, joint stiffness, and reduced grip strength, ultimately leading to impaired hand function [5].

Dynamic external fixation (DEF) utilizes the principle of ligamentotaxis by applying controlled distraction across the thumb metacarpophalangeal joint, whereby tension within the intact capsuloligamentous attachments guides displaced articular fragments toward anatomical alignment, restoring joint congruity and maintaining fracture reduction. This minimally invasive technique provides an alternative to conventional fixation methods, particularly in highly comminuted intra-articular fractures where rigid internal fixation may be technically challenging or associated with increased soft-tissue disruption [6]. This report highlights the effective use of ligamentotaxis-based DEF for managing comminuted intra-articular fractures of the thumb’s proximal phalanx base, demonstrating its value as a minimally invasive alternative when rigid fixation is unsuitable.

## Case presentation

Two male patients presented with acute right thumb injuries resulting in comminuted intra-articular fractures at the base of the proximal phalanx. The first patient, a 34-year-old male, sustained a crush injury due to a dropped dumbbell, while the second, a 35-year-old male, presented following a work-related fall from height. Both patients complained of severe pain, swelling, and deformity of the right thumb.

Radiology and diagnostic assessment

Initial radiographs in both cases demonstrated fractures involving the base of the proximal phalanx of the thumb. In Case 1, imaging obtained at an outside facility revealed a displaced fracture, which was further evaluated at our center using repeat radiographs and computed tomography (CT), confirming a comminuted intra-articular fracture with multiple fragments, loss of joint congruity, and significant displacement (Figures 1A-1C).

**Figure 1:**
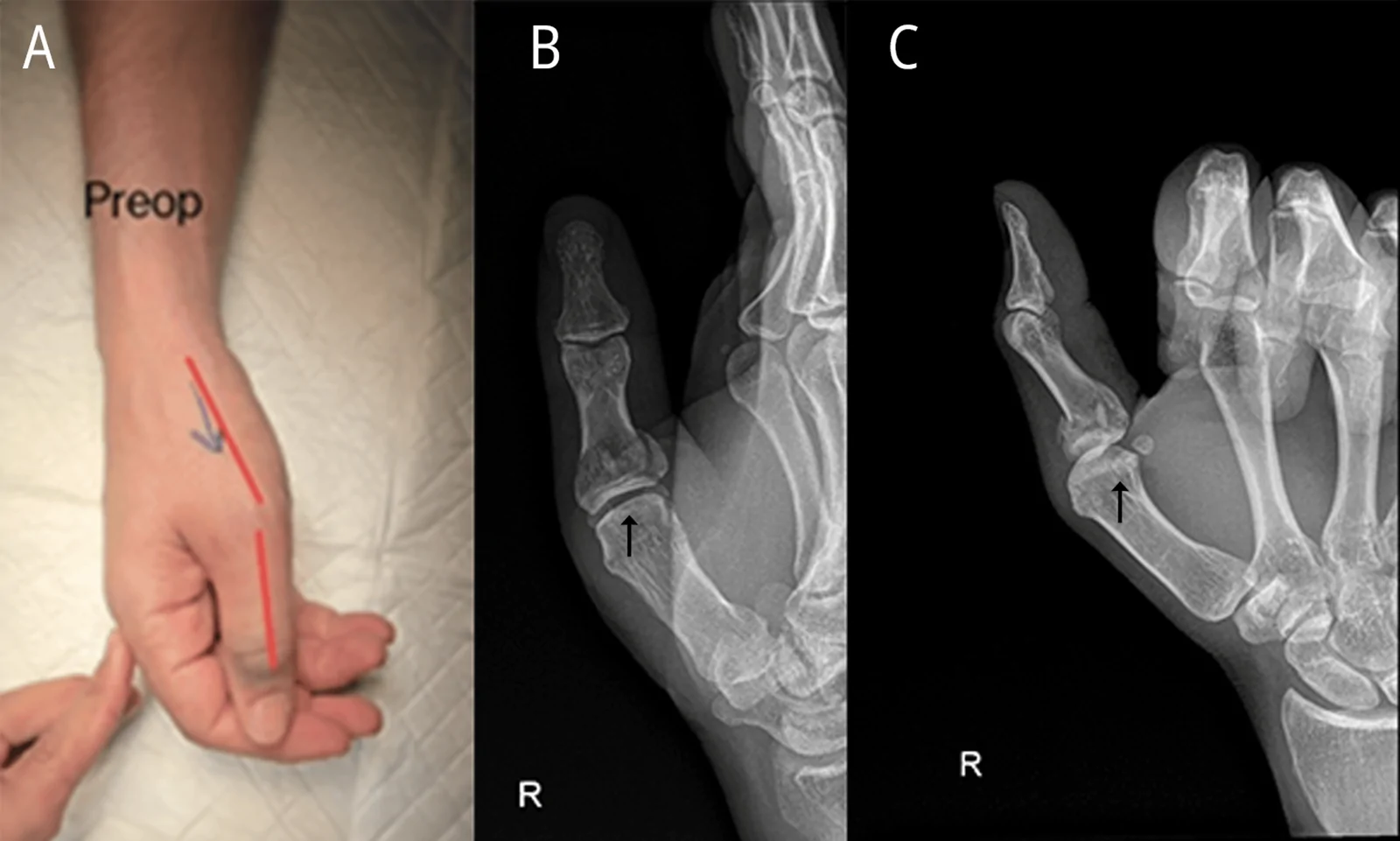
Preoperative clinical and radiographic assessment. (A) Preoperative clinical image demonstrating thumb deformity and malalignment secondary to a comminuted intra-articular fracture at the base of the proximal phalanx. (B) AP radiograph showing a comminuted intra-articular fracture of the proximal phalanx base. (C) Lateral radiograph demonstrating displacement of the proximal phalanx base fracture. Arrows indicate the site of injury.

In Case 2, CT imaging demonstrated a comminuted intra-articular fracture with approximately 40° dorsal angulation and articular incongruity. The patient initially opted for conservative management; however, follow-up radiographs showed progressive displacement and worsening deformity, necessitating surgical intervention (Figures 2A, 2B).

**Figure 2:**
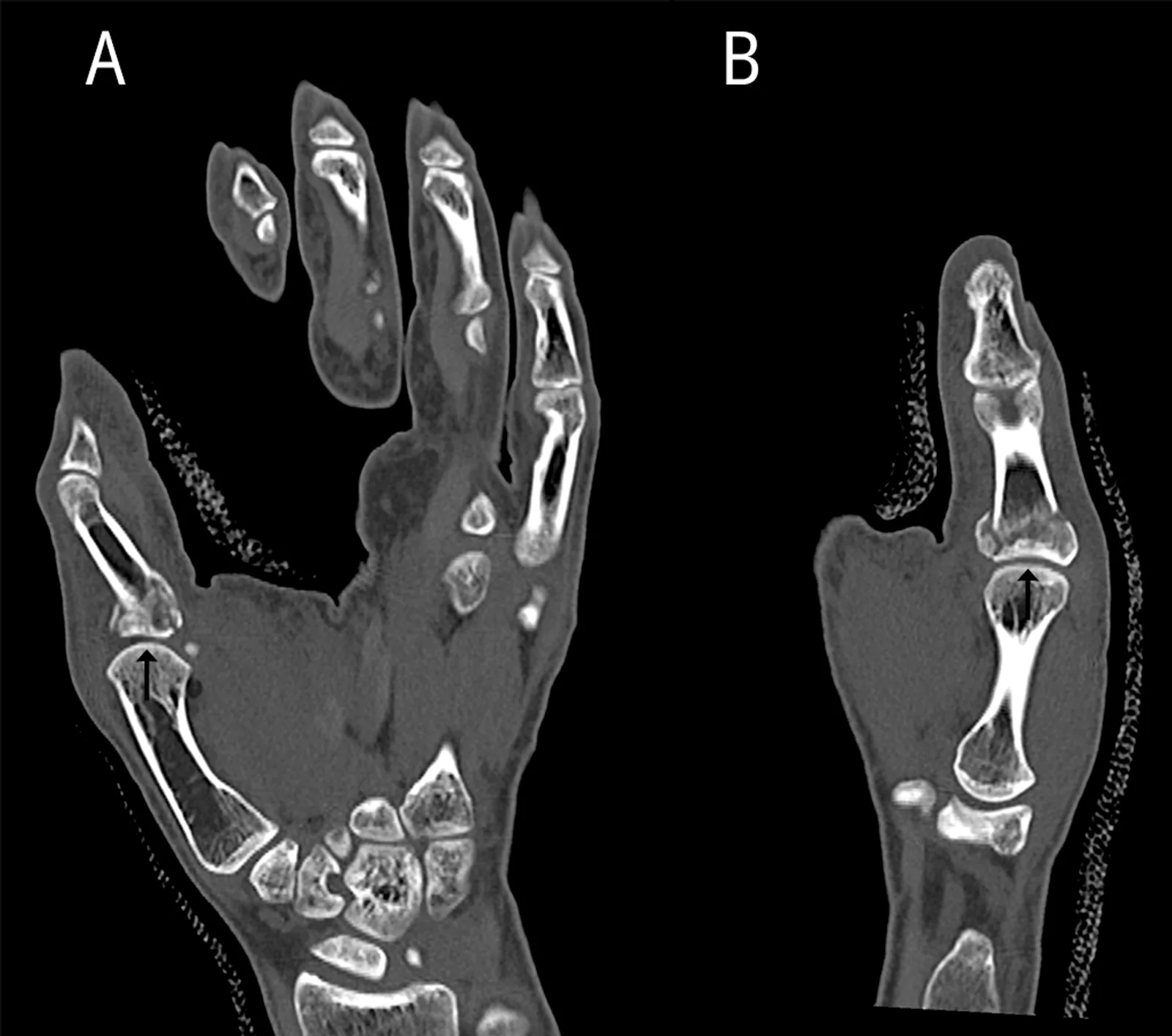
Preoperative computed tomography evaluation. (A) Sagittal CT image demonstrating fragment displacement and articular incongruity. (B) Coronal CT image showing a comminuted intra-articular fracture at the base of the proximal phalanx. Arrows indicate the site of injury.

Both patients were diagnosed with displaced comminuted intra-articular fractures involving the base of the proximal phalanx of the right thumb. In Case 2, interval progression following failed conservative management further supported the need for operative stabilization.

Surgical technique

Both patients underwent mini-open reduction and ligamentotaxis-based DEF with an operative time of approximately 1 h and a tourniquet time of 50-60 min. The patients were positioned supine under general anesthesia, and a tourniquet was applied at 200 mmHg.

Following routine preparation and draping, a mini-open dorsal incision measuring approximately 0.5 cm was made over the base of the proximal phalanx under fluoroscopic guidance. Intraoperative assessment revealed markedly displaced comminuted intra-articular fractures involving the base of the proximal phalanx. Initial reduction was performed using a mini osteotome to disimpact and realign the fracture fragments, thereby restoring bone alignment and joint congruity under fluoroscopic guidance. Following this, a Kirschner (K)-wire was introduced as a secondary step to support and maintain the reduction, assisting with elevation of the articular surface when required. Reduction was confirmed fluoroscopically, and provisional stabilization was achieved using medial and lateral K-wires (Figures 3A, 3B, 4A-4C).

**Figure 3:**
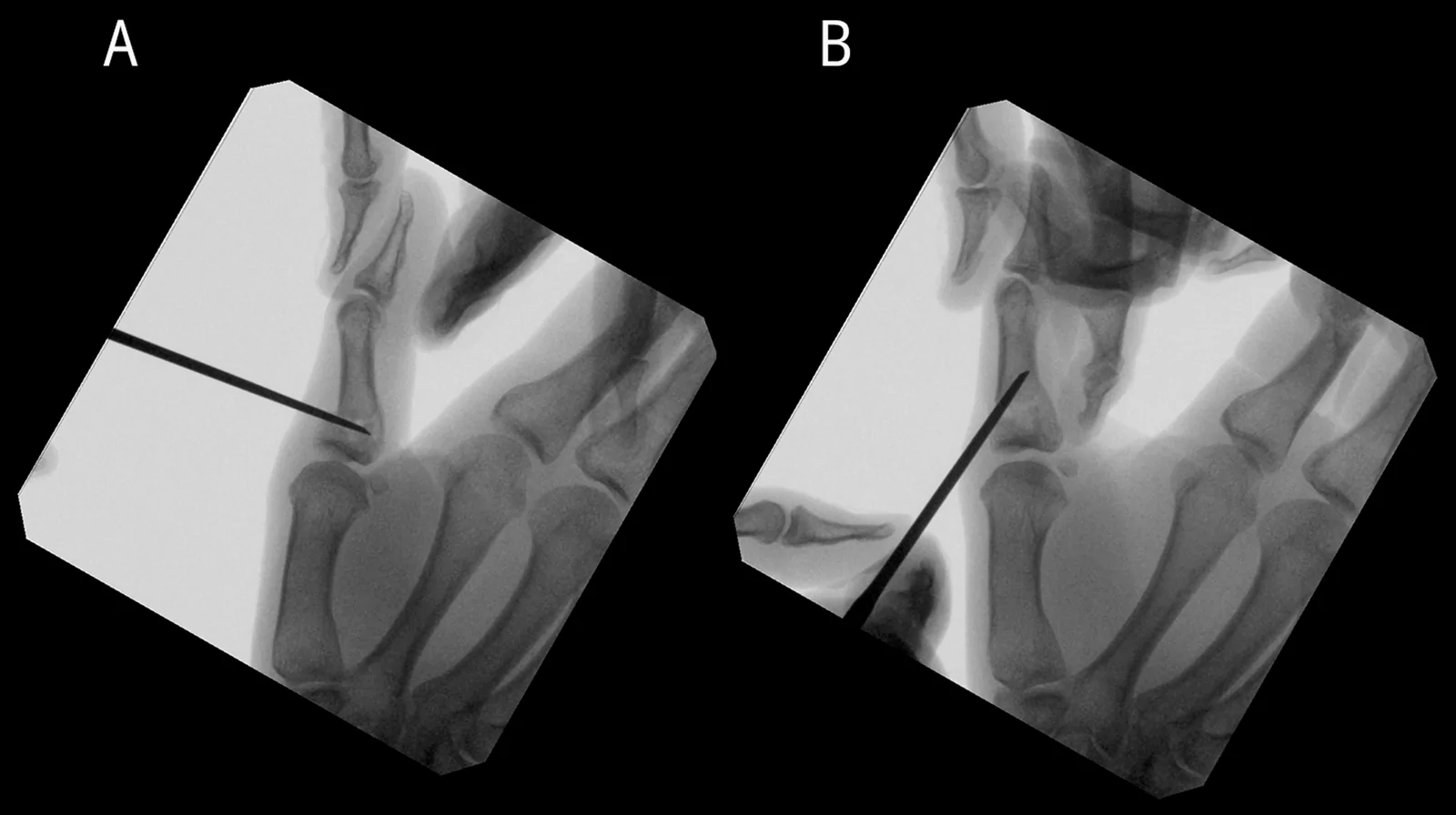
Intraoperative fluoroscopic reduction. (A) Osteotome-assisted disimpaction of the articular surface under fluoroscopic guidance. (B) Trial reduction demonstrating restoration of bony alignment and joint congruity.

**Figure 4:**
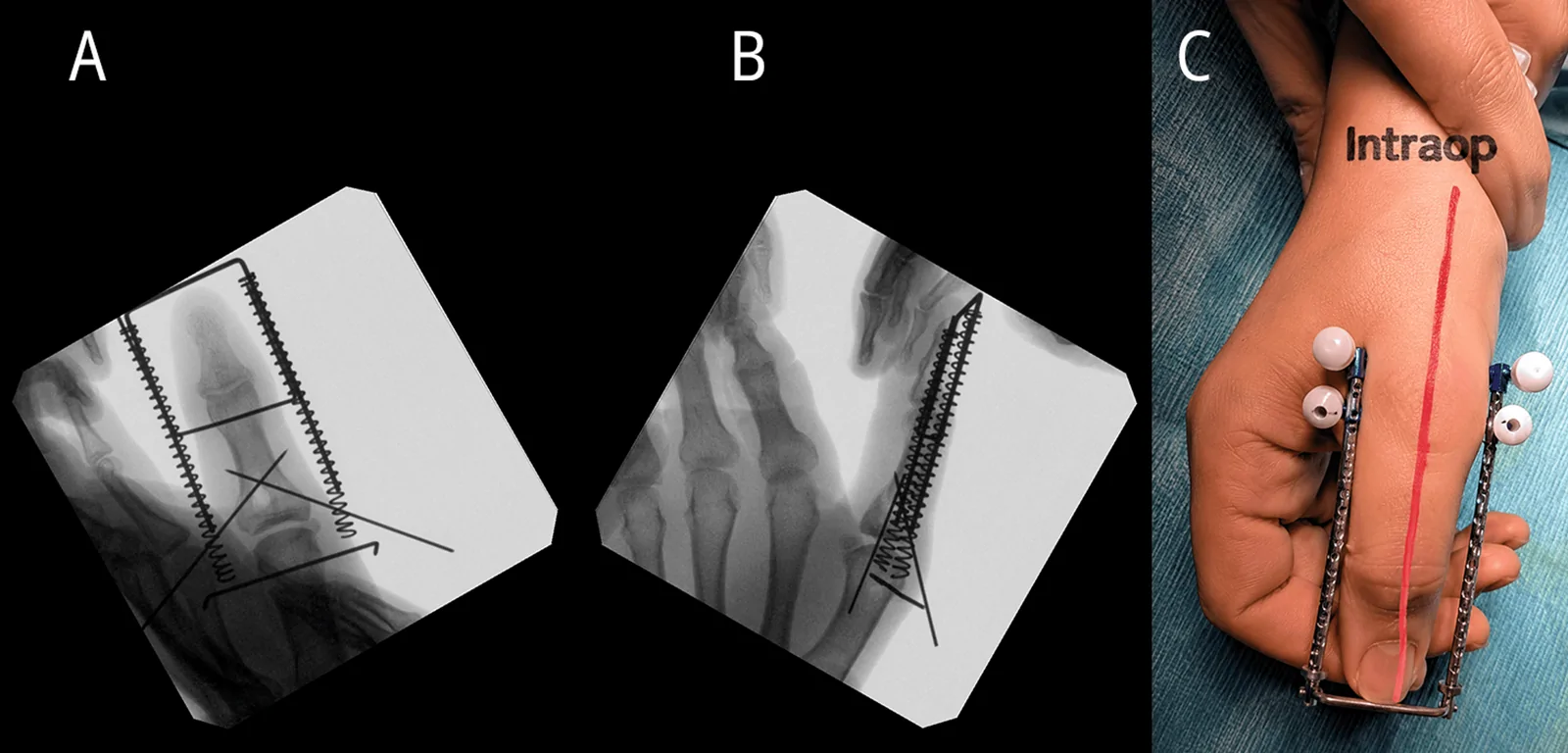
Final intraoperative C-arm and clinical image. (A, B) Radiographic images demonstrating phalangeal fractures managed with dynamic external fixation; (C) intraoperative assessment of digital alignment.

A ligamentotaxis-based DEF construct was then applied. A proximal transverse K-wire was inserted parallel to the joint line through the head of the first metacarpal, while a distal transverse K-wire was placed in the metaphyseal-diaphyseal region of the proximal phalanx. The wires were bent to 90°, connected using a spiral connector and plastic base, and tensioned to achieve controlled distraction and optimal alignment. Final reduction and fixation were confirmed under fluoroscopy, and sterile dressings were applied. No intraoperative complications were noted, and blood loss was negligible in both cases because the tourniquet remained inflated throughout the procedure.

Postoperative management

Postoperatively, both patients were admitted for monitoring of neurovascular status, pain management, and intravenous antibiotics. The operated hand was kept elevated in an arm sling during ambulation and on a pillow while resting or sleeping. Regular neurovascular assessments, including monitoring of capillary refill, were performed, and appropriate wound care was maintained. Analgesics were administered as required. Early finger range-of-motion exercises were encouraged, while use of the affected hand for weight bearing was strictly avoided.

Outcome

Postoperative recovery in both cases was uncomplicated. Pain control was satisfactory, and neurovascular status remained intact. Follow-up imaging demonstrated excellent maintenance of reduction with restoration of joint congruity. Both patients were discharged in stable condition on the first postoperative day with scheduled follow-up visits.

Functional recovery

Functional recovery was favorable for both patients. The ligamentotaxis-based DEF was removed at three weeks postoperatively, followed by gradual mobilization and progressive range-of-motion exercises. Both patients returned to work within four to five weeks (Figures 5A-5E).

**Figure 5:**
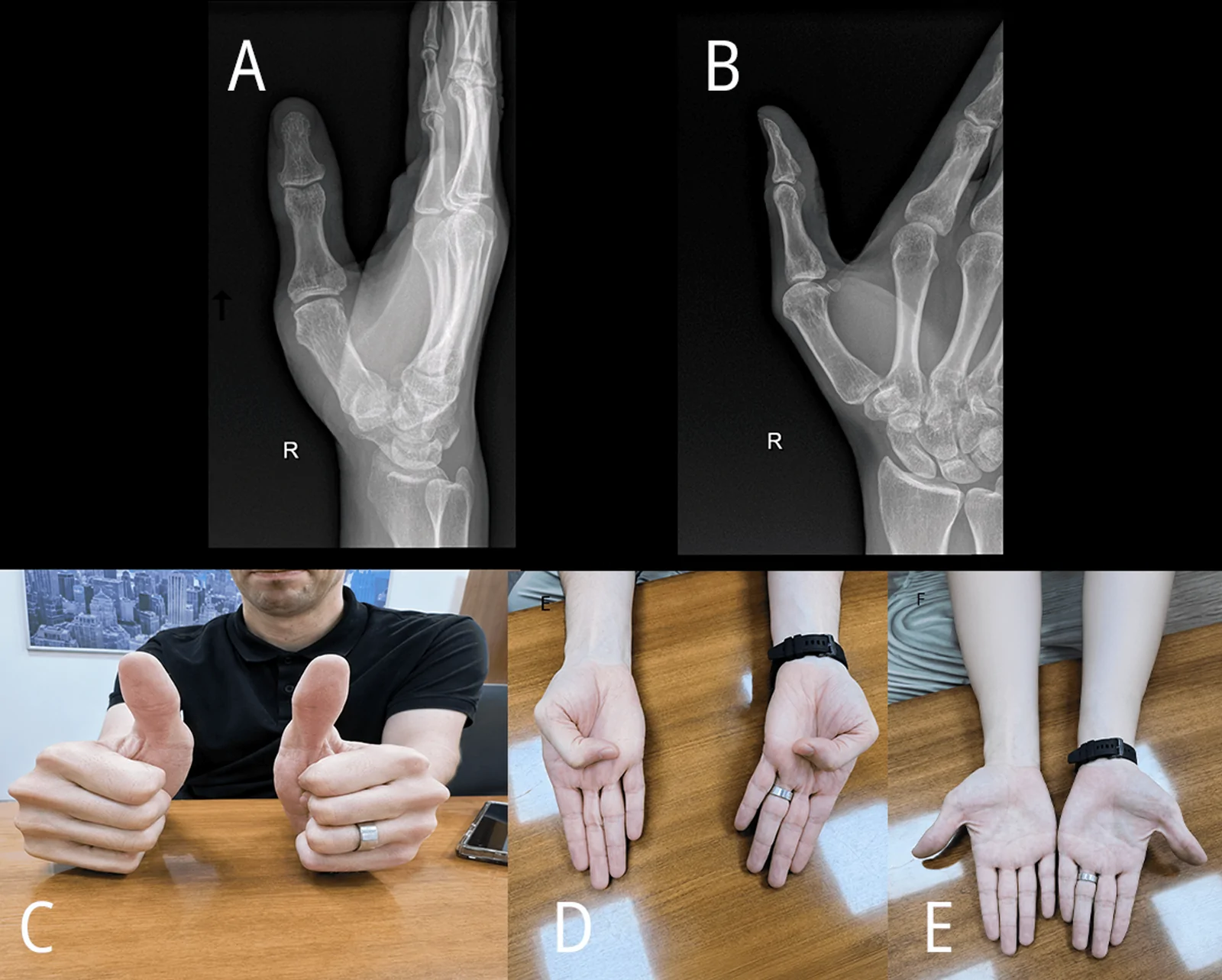
Three-month postoperative radiographic and clinical outcomes. (A) Three-month follow-up AP radiograph demonstrating fracture healing with maintained reduction and joint congruity. (B) Three-month follow-up lateral radiograph confirming preservation of alignment and articular reconstruction. (C) Clinical photograph demonstrating satisfactory thumb function at final follow-up. (D) Clinical photograph demonstrating restoration of thumb range of motion. (E) Clinical photograph demonstrating functional recovery and symmetric thumb positioning compared with the contralateral side.

At follow-up, metacarpophalangeal (MCP) joint flexion ranged from 65° to 70°, with a mild extension deficit of 3° to 5°. Grip strength recovered to approximately 90% of the contralateral side in Case 1 and 95% in Case 2. Pinch strength assessment demonstrated a key pinch of 6 kg and a tip pinch of 5 kg, indicating good functional recovery. Functional outcomes were favorable in both cases, with a Quick Disabilities of the Arm, Shoulder, and Hand (QuickDASH) score of 9.1 and a Kapandji score of 10, indicating full thumb opposition and near-complete restoration of thumb function [7,8].

## Discussion

The present findings are consistent with existing literature supporting ligamentotaxis-based dynamic external fixation (DEF) as an effective strategy for managing unstable, comminuted intra-articular phalangeal fractures, particularly those resulting from high-energy trauma with articular incongruity and a high risk of stiffness. Ahmed et al. reported favorable outcomes in a large cohort managed primarily with closed reduction and Suzuki-frame DEF, demonstrating satisfactory functional recovery despite notable complication rates, including pin-site infection (~10.3%) and joint-space narrowing (~5%) [9]. In comparison, our use of a hybrid approach incorporating mini-open reduction allowed direct manipulation and more precise restoration of articular congruity while preserving the biological advantages of ligamentotaxis, with similarly favorable functional outcomes (MCP flexion 65° to 70° and grip strength recovery of 90-95%) but without observed complications, although the small sample size warrants caution.

This concept of adapting DEF to injury complexity is further supported by Curran et al., who extended its use to more severe thumb injuries with bone loss, infection, or osteomyelitis, combining external fixation with autologous bone grafting to restore structural integrity [10]. While their focus was on reconstruction of bone stock and length, the present cases without bone loss highlight the role of DEF combined with mini-open reduction in prioritizing articular restoration and early mobilization, achieving excellent early functional outcomes including full thumb opposition (Kapandji score 10) and return to work within four to five weeks, comparable to their longer-term results (Kapandji score ≥8). Similarly, systematic evidence by Palanca et al. reinforces the effectiveness of ligamentotaxis in maintaining alignment and facilitating functional recovery in comminuted intra-articular fractures, emphasizing soft-tissue preservation through traction-based indirect reduction [6]. Although their data largely concern PIP joint fractures in other digits, the underlying principles are applicable to thumb injuries, and our addition of mini-open reduction may further enhance reduction accuracy in fractures with greater comminution.

Moreover, comparative studies such as that by Özdemir et al. demonstrate that DEF provides outcomes equivalent to open reduction and internal fixation (ORIF) in terms of union and range of motion, with lower complication rates (6.6% vs. 26.6%), supporting its role as a less invasive yet effective alternative [11]. In this context, our findings not only align with prior research but also extend it by suggesting that combining mini-open reduction with ligamentotaxis-based DEF may offer added benefits in highly comminuted thumb proximal phalanx fractures, particularly by enabling precise articular reconstruction, minimizing soft-tissue disruption, and facilitating earlier rehabilitation through shorter fixation duration. However, these findings should be interpreted with caution given the small sample size of two cases, which limits generalizability, and larger studies are needed to validate these observations.

## Conclusions

This report highlights the potential effectiveness of ligamentotaxis-based dynamic external fixation (DEF) in managing complex, high-energy intra-articular fractures of the base of the proximal phalanx of the thumb. In both patients, a simple ligamentotaxis-based DEF construct enabled controlled distraction, restoration of joint congruity, and stable alignment while minimizing soft-tissue disruption. Both cases demonstrated uneventful recovery, preserved neurovascular function, and favorable postoperative radiographic outcomes. These findings support DEF as a reliable, minimally invasive option for highly comminuted thumb fractures unsuitable for closed reduction or conventional open reduction and internal fixation, with favorable early functional and radiological outcomes.
